# Evaluating the effects of Danhong injection in treatment of acute ischemic stroke: study protocol for a multicenter randomized controlled trial

**DOI:** 10.1186/s13063-015-1076-4

**Published:** 2015-12-09

**Authors:** Bing Li, Yilong Wang, Jingjing Lu, Jun Liu, Ye Yuan, Yanan Yu, Pengqian Wang, Xingquan Zhao, Zhong Wang

**Affiliations:** Institute of Basic Research in Clinical Medicine, China Academy of Chinese Medical Sciences, 16 Nanxiaojie, Dongzhimennei, Beijing, 100700 China; Institute of Information on Traditional Chinese Medicine, China Academy of Chinese Medical Sciences, 16 Nanxiaojie, Dongzhimennei, Beijing, 100700 China; Beijing Tiantan Hospital Affiliated to Capital Medical University, 6 Tiantan Xili, Dongcheng District, Beijing, 100050 China; Changzhou TCM Hospital, Heping North Road, Changzhou, Jiangsu 213004 China

**Keywords:** Acute ischemic stroke, Danhong injection, Chinese medicine, Randomized controlled trial

## Abstract

**Background:**

Danhong injection (DHI) has been widely prescribed to patients with acute ischemic stroke (AIS). However, due to methodological deficiencies, previous research has not yet provided rigorous evidence to support the use of DHI in the treatment of AIS. Therefore, we designed this multicenter, randomized, controlled, and double-blind trial to evaluate the efficacy and safety of DHI for AIS.

**Methods/Design:**

It is a randomized, multicenter, double-blind, placebo-controlled, adaptive clinical trial. A total of 864 eligible patients will be randomized into either the DHI or placebo group in a 2:1 ratio. All patients will be given the standard medical care as recommended by guidelines. Participants will undergo a 2-week treatment regimen and 76-day follow-up period. The primary outcome is the proportion of patients with a favorable outcome, defined as a score of 0–1 on the modified Rankin scale at day 90. Secondary outcomes include a change in the total score of the Chinese medicine symptom scales of “*Xueyu Zheng*” (blood stasis syndrome), the proportion of patients with a Barthel Index score of ≥90, the proportion of patients with an improvement in NIHSS score of ≥4 or NIHSS score of 0–1, quality of life measured by the EQ-5D scale, etc. Safety outcomes such as global disability (mRS ≥3) at day 90 will also be assessed. The changes in mRNA and microRNA profiles in 96 patients selected from certain centers will also be assessed. As this is an adaptive design, two interim analyses are prospectively planned, which will be carried out after one-third and two-thirds of patients have completed the trial, respectively. Based on the results of the interim analyses, the Data Monitoring Committee (DMC) will decide how to modify the study.

**Discussion:**

This trial will provide high-quality evidence for DHI in treatment of AIS.

**Trial registration:**

Clinical Trials.gov NCT01677208 (Date of registration 22 December 2012).

**Electronic supplementary material:**

The online version of this article (doi:10.1186/s13063-015-1076-4) contains supplementary material, which is available to authorized users.

## Background

Acute ischemic stroke (AIS), characterized by an occlusion of the cerebral artery, is one of the leading causes of mortality, long-term disability, and morbidity. Thrombolytic therapy with intravenous tissue plasminogen activator (tPA), which remains the only therapeutic drug for AIS approved by the US Food and Drug Administration, is limited by the narrow time window of thrombolysis, bleeding complications, or high costs [1]. Other treatment strategies mainly utilize therapeutic agents to prevent or reduce cell damage from ischemia [2]. Hundreds of neuroprotective agents for ischemic stroke have reached clinical trials, but no optimal drugs for the clinical treatment of AIS have yet been developed [3]. This leads us to search for novel therapeutic approaches, and to investigate whether natural compounds extracted from herbs may be an alternative option to improve treatment for AIS [4].

Danhong injection (DHI), extracted from *Radix Salviae miltiorrhizae* (Danshen) and *Flos Carthami tinctorii* (Honghua), is a Chinese medicinal product approved by the China Food and Drug Administration (CFDA), and it is widely used in China for treating AIS [5]. Previous studies demonstrated the neuroprotective efficacy of DHI in rat models with cerebral ischemic-reperfusion injury [6, 7], and that the underlying pharmacological mechanisms might involve anti-inflammatory, anticoagulant, antithrombotic, antifibrinolytic, and antioxidant activities [8]. A number of clinical studies also supported the efficacy and safety of DHI in the management of AIS, but due to the methodological deficiencies of inadequate randomization, no double blinding, no placebo control, or incomplete outcome data, the quality of these studies was assessed as generally low [9]. There is a lack of critically appraised evidence such as well-designed randomized controlled trials (RCTs) to justify its clinical use and recommendation. Thus, we aim to investigate the efficacy and safety of DHI in the treatment of AIS in a well-designed, randomized, multicenter, double-blind and placebo-controlled trial. The trial was registered with an identifier (NCT01677208) in Clinical Trials.gov.

## Methods/Design

### Design

**Fig. 1:**
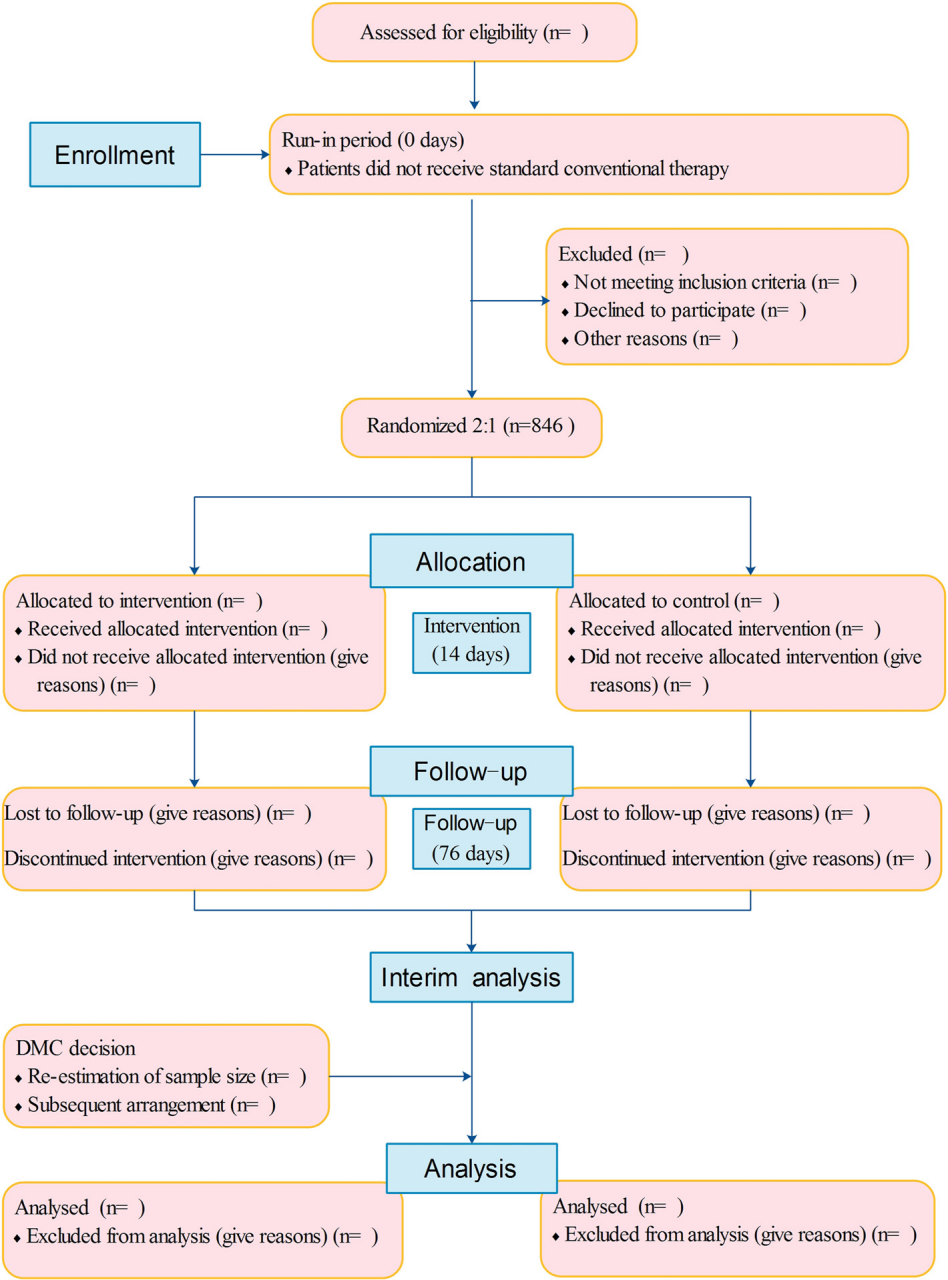
The flow diagram of this trial

This is an adaptive design, multicenter, randomized, double-blind, placebo-controlled, superiority trial of DHI in the treatment of acute ischemic stroke. Eligible patients are randomly assigned in a 2:1 ratio into the DHI group or placebo control group (Fig. 1, Table 1). An adaptive design will be applied; if the superiority of DHI is observed at the time of the interim analysis, the trial can be terminated early.

**Table 1:** Data collection schedule and measures

Period	Run-in period	Treatment period		Follow-up period		
Day	0	7 ± 2	14 ± 2	30 ± 5	60 ± 5	90 ± 3
Informed consent	×					
Inclusion/exclusion criteria	×					
Medical history	×					
UCG	×					
TOAST	×					
Medical examination	×	×	×	×	×	×
Concomitant medications	×	×	×	×	×	×
Outcomes						
BI scale	×	×	×	×	×	×
mRS scale	×	×	×	×	×	×
NIHSS	×	×	×	×	×	×
Chinese medicine symptom scales of “*Xueyu Zheng*”	×	×	×	×	×	×
EQ-5D scale	×	×	×	×	×	×
Global disability on mRS						×
New-onset major vascular events						×
Overall mortality						×
Incidence of severe hemorrhages						×
Incidence of moderate hemorrhages						×
AEs and SAEs						×
Profiles of microRNA in 60 patients from certain centers	×		×			×
Profiles of mRNA in 60 patients from certain centers	×		×			×

*UCG* ultrasound cardiogram, *TOAST* Trial of Org 10 172 in Acute Stroke Treatment, *BI* Barthel Index, *mRS* modified Rankin Scale, *NIHSS* National Institutes of Health Stroke Scale, *AEs* adverse events, *SAEs* serious adverse events, *microRNA* micro-ribonucleic acid, *mRNA* messenger ribonucleic acid

### Participant eligibility

To be fully eligible for participation in the trial, acute ischemic stroke inpatients must meet all of the following inclusion criteria and not meet any of the following exclusion criteria.

#### Inclusion criteria

1. Female or male inpatients.
2. Aged 18–70 years.
3. Clinical diagnosis of ischemic stroke causing a measurable neurological deficit defined as impairment of language, motor function, cognition and/or gaze, vision or neglect. Ischemic stroke is defined as an event characterized by the sudden onset of an acute focal neurologic deficit presumed to be due to cerebral ischemia after computed tomography (CT) scan excludes hemorrhage.
4. Clinical diagnosis of “*Xueyu Zheng*” (blood stasis syndrome) defined as a total score of Chinese medicine symptom scales of “*Xueyu Zheng*” in ischemic stroke ≥20. The Chinese medicine symptom scales of “*Xueyu Zheng*” include the visual analogue scales (VAS) for the following items: (1) hemiplegia (0–10), (2) numbness of limbs (0–10), (3) dark face (0–9), (4) purple or dark lip (0–8), (5) rough skin (0–4), (6) pain with fixed point (0–5), (7) purple or dark tongue (0–10), (8) ecchymosis on the tongue (0–10), (9) purple sublingual vessels (0–10), (10) sublingual varices (0–8), (11) unsmooth pulse (0–8), and (12) intermittent pulse (0–1).
5. Onset of symptoms within 1 week prior to initiation of study drug administration.
6. Clinical diagnosis of cerebral anterior circulation obstruction.
7. 4 ≤ National Institutes of Health Stroke Scale (NIHSS) score <20.
8. Patients willing to participate voluntarily and to sign a written informed consent document. Informed consent will be obtained from each patient or the subject’s legally authorized representative or relatives, or deferred where applicable, according to the regulatory and legal requirements of the participating centers.

#### Exclusion criteria

1. Evidence of intracranial hemorrhage (ICH) or other cerebral diseases (e.g., vascular malformation, tumor, abscess or multiple sclerosis) on the CT or magnetic resonance imaging (MRI) scan.
2. Patients who underwent thrombolysis or endovascular treatment.
3. Known history of allergy or suspected allergy to the study drug.
4. Fasting blood glucose <2.8 or >16.8 mmol/l or with severe complications due to diabetes (e.g., peripheral neuropathy, or diabetic gangrene).
5. Liver function impairment with the value of alanine aminotransferase (ALT) or aspartate aminotransaminase (AST) over 1.5-fold the upper limit of normal range.
6. Renal dysfunction with the value of serum creatinine over 1.5-fold the upper limit of normal range.
7. Severe cardiac dysfunction on echocardiogram or the New York Heart Association (NYHA) heart functional class III or IV.
8. History of prior stroke with a modified Rankin Scale (mRS) score ≥2.
9. Atrial fibrillation complications.
10. Severe stroke as assessed by appropriate imaging techniques (e.g., massive cerebral infarction affecting more than one lobe of the brain or over one-third of the blood-supply area of the middle cerebral artery).
11. Legally disabled patients.
12. Hemorrhagic tendency or severe or dangerous bleeding within the prior 3 months.
13. Suspected alcohol addiction or drug abuse.
14. Severe complications that would make the condition more complicated as assessed by the investigator.
15. Women who are pregnant, lactating or have a positive pregnancy test, or women who plan to become pregnant in the subsequent 6 months.
16. Women who have a menstrual period at baseline.
17. Patients who are participating in other trials or have participated in other trials within the prior 3 months.

### Recruitment

Recruitment into the trial started in December 2012 and is estimated to end in December 2015. Patients with AIS will be screened for eligibility according to the inclusion and exclusion criteria. Patients will be thoroughly informed of the details of the study and its potential benefits and risks. Only those who meet the inclusion criteria and willingly agree to provide written informed consent will be included. This study will be conducted in 43 hospitals across China, and the number of hospitals could be increased after the interim analysis.

### Sample size estimation

The proportion of patients who had a favorable outcome, defined as a modified Rankin Scale (mRS) score of 0–1, after standard medical care coupled with placebo or DHI for 90 days was unclear. According to previous studies [10, 11], the proportion of patients who had a mRS score of 0–1 was about 28.7–45.2 % when only treated with placebo, so we estimate the proportion is 45 % when placebo coupled with standard medical care are used. In this trial, it is hypothesized that an increase of at least 10 % is of clinical significance for the DHI group. An adaptive design was selected and EAST5.2 software was used to calculate the statistical sample size. After calculation, the number of subjects was initially estimated to be 703 (one-sided test, α = 0.05, β = 0.2). To allow for a dropout rate of 20 %, a total of 846 patients will be recruited. According to the adaptive design, the sample size may be adjusted after the interim analyses, which will be carried out when one-third (282) and two-thirds (564) of patients have completed the trial, respectively. With a 2:1 randomization ratio, the number of participants in the DHI group is 564 and that in the control group is 282.

### Randomization

All participants will be randomized into the DHI intervention group or placebo control group. The randomization procedure will be performed by the central online randomization system.

### Blinding

All patients, treating physicians and statisticians will be blinded to group assignment until the end of this trial. Considering the different colors of DHI and 0.9 % saline, the dropping bottles will be wrapped in sealed shaded bags (that cannot be unwrapped during infusion; the integrity of these bags will be checked after infusion) and brown infusion devices will be used to conceal the liquid color. Two full-time nurses who cannot contact each other will implement the above procedures independently. One nurse will be responsible for the drug preparation and sealing the dropping bottles with the shaded brown bags in a designated transfusion room; while the other nurse will bring the prepared drugs from the transfusion room to the infusion nurse and supervise the infusion process to ensure the drug allocation is blinded to patients. A confidentiality agreement will be signed by the two full-time nurses before study initiation.

### Interventions

Participants in both intervention and control groups will be provided with standard medical care throughout the trial, which should be in strict accordance with the China Guideline for the Diagnosis and Treatment of Acute Ischemic Stroke (2010) [12], including medical care for vital signs, control of temperature, blood pressure and glucose, improving cerebral blood circulation, antiplatelet treatment and nutritional supportive care. All of these routine treatments will be recorded in patients’ medical records as well as their case report forms (CRFs) in detail. Any concomitant medications that are necessary to manage any serious underlying disease should also be recorded in patients’ medical records and their CRFs. However, any other Chinese herbal medicines or Chinese patent medicines with the similar pharmacological effect of DHI will not be allowed throughout the study period.

#### Intervention group

In addition to standard medical care, participants in the intervention group will be treated with DHI (40 ml, once daily) plus 0.9 % normal saline (250 ml, intravenously, once daily). The infusion rate should be 20–40 drops/min, and the infusion must be completed within 3 hours. DHI cannot be mixed with other liquids to be infused.

#### Control group

In addition to standard medical care, participants in the placebo group will be treated with the DHI placebo (0.9 % normal saline, 40 ml, once daily) plus 0.9 % normal saline (250 ml, intravenously, once daily). The infusion rate should be 20–40 drops/min, and the infusion must be completed within 3 hours. The DHI placebo should be prepared by the full-time nurse.

### Outcome measures

#### Primary outcome

The primary outcome is the proportion of patients with a favorable outcome, defined as a score of 0–1 on the mRS [13] at day 90. The mRS is a commonly used scale for measuring the degree of disability or dependence in the daily activities of people who have suffered a stroke or other causes of neurological disability. The scale runs from 0–6, ranging from perfect health without symptoms to death.

#### Secondary outcomes

1. The total score of Chinese medicine symptom scales of “*Xueyu Zheng*” (blood stasis syndrome).
2. The proportion of patients with the Barthel Index (BI) score of ≥90. BI uses ten variables to describe the activities of daily living (ADL) and mobility, and a higher number is associated with a better performance of ADL [14].
3. The proportion of patients with an improvement in the NIHSS score of ≥4 or the NIHSS score of 0–1. NIHSS is a tool used to objectively quantify the impairment caused by stroke. A NIHSS score of 0 typically indicates normal function in that specific ability, while a higher score is indicative of some level of impairment [15].
4. The quality of life measured by the EQ-5D scale. The EQ-5D scale has five standard dimensions to measure the generalized health-related quality of life [16].
5. Global disability (mRS ≥3) at day 90.
6. Incidence of new-onset major vascular events within 90 days. Major adverse vascular events include ischemic stroke, hemorrhagic stroke, transient ischemic attack, myocardial infarction and vascular-related death.

The safety of DHI is evaluated by the incidences of global disability (mRS ≥3) at day 90, new-onset major vascular events, severe hemorrhages and moderate hemorrhages within 90 days. The definition of “severe hemorrhages” and “moderate hemorrhages” is in accordance with the GUSTO bleeding criteria [17]. Other safety measures include the overall mortality at day 90. All the adverse events (AEs) and serious adverse events (SAEs) within 90 days should be observed and documented. Other outcome measures include the changes in the mRNA and microRNA profiles in 60 patients selected from certain centers. An overview of the measurement data collection schedule is shown in Table 1.

### Interim analysis

As an adaptive trial, two interim analyses will be carried out in a blinded manner after one-third and two-thirds of patients have completed the trial, respectively. Based on the statistical results of the interim analyses, the Data Monitoring Committee (DMC) will decide on the re-estimation of the sample size and subsequent modifications to the trial protocol. As the two interim analyses may lead to an increased possibility of type 1 error, a Lan-DeMets alpha spending function with an O’Brien-Fleming boundary will be used to adjust the results.

### Safety and adverse events

AEs are undesirable experiences occurring to participants during the trial, whether or not they are considered to be related to the experimental treatment. All AEs during the study must be recorded on the CRF, including the nature of each event, time and date of onset, duration, intensity, seriousness criteria, an assessment of its cause, the need for specific therapy, the actions taken and its outcome. According to the severity of AEs, the investigator will determine whether the participant should be withdrawn from the study, and follow-up procedures should be performed and recorded in detail. The relationship between the experimental treatment and the AEs should be assessed and recorded by the investigator.

A SAE is defined as any untoward occurrence or effect that causes death, is life-threatening, requires prolonged hospitalization, results in persistent significant disability, or leads to a congenital anomaly or birth defect. If a SAE occurs, the investigator must immediately report it to the principal investigator and the ethics committee, and record it on the CRF with a signature and date. The classification of the severity of AEs and relationship to the studied drug are conducted according to our previous study [18].

### Data collection and management

An overview of study visits and data collection schedule of this study is shown in Table 1. All data will be recorded by trained clinical investigators in a standardized electronic case report form (e-CRF) via the Brightech-Magnsoft Clinical Information Management Systems (CIMS, Brightech-Magnsoft Inc., Somerset, NJ, USA). To ensure the accuracy and reliability of the data, the study monitor will verify and cross-check the eCRFs against the investigator’s source document records and drug-dispensing log. In case of any discrepancies in the cross-check procedure, the results will be sent to the investigator for resolution. Except for the treatment code, any individual identification of the subjects will not be released until the database is closed.

To guarantee the validity and integrity of the trial design, three committees have been established by the multicenter trial coordination group. The first is the Clinical Trial Guidance Committee, which is responsible for the study design and the implementation process. The second is the DMC (also known as DSMB, Data and Safety Monitoring Board), which will supervise the data collection process in order to control its quality. The third is the Outcome Evaluation Committee (OEC), which will evaluate the key outcomes (including outcome measurements and AEs) based on clinical expertise.

### Statistical analysis

Statistical analysis will be performed by the Statistical Analysis System (SAS, Version 9.1, SAS Institute Inc., Cary, NC, USA). Except for the microRNA and mRNA profiles, most outcome measurements will be analyzed using full analysis sets (FAS) and per protocol sets (PPS), according to intention-to-treat (ITT). Compliance analysis will be based on the FAS, and analysis of concomitant medications will be based on the safety set (SS). Analytical statistics to estimate the difference in the number of participants who have completed or been withdrawn from the trial between groups will be calculated. Baseline characteristics will be summarized by means of simple descriptive statistics. For parametric tests, the results will be described as mean (standard deviation), maximum, minimum, and median, and nonparametric test median (quartile deviation). Categorical data will be described by absolute values and proportion.

Enumeration data will be analyzed by chi-square test (χ^2^ test) test, Cochran-Mantel-Haenszel (CMH) χ^2^ test, Fisher’s exact test, or Wilcoxon rank test. Quantitative data will be compared by analysis of variance (ANOVA) and *t* test. Two-sample *t* tests will be employed for comparison between groups. Paired *t* test will be used to analyze significant difference between pre- and post-treatment. Student-Newman-Keuls (SNK) significance test will be used for pairwise comparison. Chi-squared test will be used for nominal categorical data, rank sum test for ordinal categorical data. The analysis of covariance (ANCOVA) will be used to control potential confounding variables. In all analyses, statistical uncertainty will be expressed by means of 95 % CI.

#### Efficacy analysis

Primary and secondary efficacy measures will be analyzed. Any factors impacting efficacy should be taken into account as covariance, such as age and gender. The ANCOVA model, Cox’s proportional hazards regression model, or logistic regression model will be used to assess treatment effects.

#### Safety analysis

Safety will be analyzed in terms of the incidence of new-onset major vascular events within 90 days, overall mortality within 90 days, incidence of severe hemorrhages within 90 days, incidence of moderate hemorrhages within 90 days, as well as AEs and SAEs. Safety analysis will be done in the safety set (SS), defined as a subset of subjects who were randomized and received at least one treatment.

### Ethical considerations

This trial will be conducted in adherence to the Declaration of Helsinki (Edinburgh 2000). Informed consent will be obtained from all participants or their legal representative, in writing, before inclusion in the trial. The trial has been approved by the local institutional ethics committees (the Ethics Committee of the Institute of Basic Clinical Research, China Academy of Chinese Medical Sciences; the Ethics Committee of Chinese PLA General Hospital). The ethics committees of the clinical centers which approved this study are listed in Additional file 1.

## Discussion

Although a number of trials on DHI in the treatment of AIS have been published, the methodological deficiencies of these trials such as inadequate randomization, no double blinding, no placebo control, and incomplete outcome data may have lead to various biases, so there is still a lack of robust evidence to prove its efficacy. It is widely accepted that a well-designed RCT is the gold standard for evaluating the clinical efficacy and safety of Chinese medicine (CM). To the best of our knowledge, this study is the first rigorously designed RCT to evaluate the efficacy and safety of DHI for AIS. In this trial, a central online randomization system and triple-blind method are used to avoid the potential selection and detection biases. Although there are limitations of placebo control in CM research due to the unique characteristics of herbal medicines or CM such as color, appearance, odor and properties, the placebo control in this study will be strictly implemented. The wrapped dropping bottles and brown transfusion devices will be used to facilitate the placebo control, making the treatment seemingly identical between the DHI and placebo groups, so the potential placebo effect will be excluded. Besides, an adaptive design, which includes a prospectively planned opportunity for modification of the trial design, has been adopted to improve the efficiency of the study (e.g., shorter duration, or fewer patients) [19]. Moreover, the add-on study design, which can be summarized as “A + B versus B” model, will also be applied in this trial. The routine treatment will be administered in both the DHI and placebo groups, so as to provide reliable evidence for DHI as an effective add-on therapy for patients with AIS.

Both doctor-reported outcome (DRO) and patient-reported outcome (PRO) models will be used as outcome measures in this study. In addition to the doctor’s observation, patient-centered outcomes focusing on patient’s quality of life and relief of symptoms will also be applied. It is believed that CM may demonstrate superiorities in terms of alleviating symptoms and improving quality of life. In traditional Chinese medicine (TCM), disease treatments are based on syndrome (*Zheng* in Chinese) differentiation, and improvement of the syndrome often indicates better prognosis of the disease. It is proposed that blood stasis syndrome (*Xueyu Zheng*) is the most common type of AIS [20]. Therefore, the TCM syndrome score is used as an outcome measure, which mainly focuses on the improvement of patient symptoms. DHI is an approved Chinese medicinal product extracted from *Radix Salviae miltiorrhizae* and *Flos carthami*, which can produce a remarkable curative effect on *Xueyu Zheng*. Several studies have validated the vascular-protective effects of DHI [21–24]. Based on the theoretical and clinical evidence, DHI may have a great therapeutic value in real-world clinical practice.

There are also some limitations in this trial. First, because of the relatively short follow-up period, it is not possible to assess the long-term effects of DHI on the primary outcome, and the potential role of DHI for reducing major vascular events and overall mortality in the longer term will remain unknown. Second, due to the absence of a “head-to-head” design, this study can only demonstrate the complementary effect of DHI, but still cannot draw a definite conclusion regarding whether DHI can serve as an alternative therapy for AIS. Therefore, well-designed RCTs comparing DHI versus conventional anti-ischemia therapy with a longer-term follow-up are warranted in the future.

## Trial status

The study began recruiting patients in 2012. The trial is currently recruiting patients.

## Additional file

Additional file 1:
**The ethical bodies that approved this study in the various clinical centres.** (DOCX 15 kb)

## Abbreviations

ADL: Activities of daily living
AEs: Adverse events
AIS: Acute ischemic stroke
ALT: Alanine aminotransferase
ANOVA: Analysis of variance
ANCOVA: Analysis of covariance
AST: Aspartate aminotransaminase
BI: Barthel Index
CI: Confidence interval
CM: Chinese medicine
CRFs: Case report forms
CT: Computed tomography
DHI: Danhong injection
DMC: Data Monitoring Committee
DRO: Doctor-reported outcome
EQ-5D: EQ-5D scale
FAS: Full analysis set
ICH: Intracranial hemorrhage
ITT: Intention-to-treat
MRI: Magnetic resonance imaging
mRNA: Messenger ribonucleic acid
microRNA: Microribonucleic acid
mRS: Modified Rankin Scale
NIHSS: National Institutes of Health Stroke Scale
OEC: Outcome Evaluation Committee
PPS: Per protocol sets
PRO: Patient-reported outcome
RCT: Randomized controlled trial
SAEs: Serious adverse events
SS: Safety set
TCM: Traditional Chinese medicine
TOAST: Trial of Org 10172 in Acute Stroke Treatment
tPA: Tissue plasminogen activator
UCG: Ultrasound cardiogram
VAS: visual analogue scale
