# Supplementary material for: Evaluating the effects of Danhong injection in treatment of acute ischemic stroke: study protocol for a multicenter randomized controlled trial
Source: Trials. 2015 Dec 9;16:561. doi: 10.1186/s13063-015-1076-4 (PMC4673756; doi:10.1186/s13063-015-1076-4)
Supplement: Additional file 1: — The ethical bodies that approved this study in the various clinical centres. (DOCX 15 kb) [file 13063_2015_1076_MOESM1_ESM.docx]

**Evaluating the effects of Danhong** **injection in treatment of acute ischemic stroke: study protocol for a multicenter randomized controlled trial**

Bing Li^1,2^, Yilong Wang^3^, Jingjing Lu^3^, Jun Liu^1^, Ye Yuan^4^, Yanan Yu^1^, Pengqian Wang^1^, Xingquan Zhao^3^*, Zhong Wang^1^*

1. Institute of Basic Research in Clinical Medicine, China Academy of Chinese Medical Sciences, 16 Nanxiaojie, Dongzhimennei, Beijing 100700, China

2. Institute of Information on Traditional Chinese Medicine, China Academy of Chinese Medical Sciences, Beijing 100700, China

3. Beijing Tiantan Hospital Affiliated to Capital Medical University, No. 6, Tiantan Xili, Dongcheng District, Beijing, 100050, China.

4. Changzhou TCM Hospital, Heping North Road, Changzhou 213004, Jiangsu, China.

**The ethical bodies that approved this study in the various centres involved.**

1. IRB of Beijing Tiantan Hospital Affiliated ti Capital Medical University
2. IRB of Peking University First Hospital
3. IEC of The First Affiliated Hospital of Tianjin University of Traditional Chinese Medicine
4. Ethics Committee of the Second Artillery General Hospital of Chinese People’s Liberation Army
5. IEC of Peking University People’s Hospital
6. IEC of Xuanwu Hospital Capital Medical University
7. Ethics Committee of Xiangya Hospital of Central-South University
8. IEC of the First Afflicted Hospital of Henan University of TCM
9. IEC of Renmin Hospital of Wuhan University
10. IEC of Zhongnan Hospital of Wuhan University
11. IEC of the Second Hospital of Jilin University
12. IEC of the Second Hospital of Shanxi Medical University
13. Ethics Committee of Hubei Zhongshan Hosipital of Drug Clinical Trial
14. IEC of Changsha Central Hospital
15. Ethics Committee of Hubei Province Hospital of Traditional Chinese Medicine
16. Ethics Committee of Chongqing Three Gorges Cental Hospital
17. Ethics Committee of Shanxi Province Hospital of Traditional Chinese Medicine
18. IEC of the Second Affiliated Hospital of Nanjing Medical University
19. Ethics Committee of Nanjing Hospital of TCM
20. IEC of the First Hospital of Changsha
21. IEC of the Second Affiliated Hospital of Zhejiang University of Traditional Chinese Medicine
22. IEC of Nanjing Drum Tower Hospital, The Affiliated Hospital of Nanjing University Medical School
23. Sichuan Regional Ethics Review Committee on Traditional Chinese Medicine
24. IEC of Zhejiang Provincial People’s Hospital
25. IEC of the First Affiliated Hospital of Zhejiang University of Traditional Chinese Medicine
26. Ethics Committee of Brains Hospital of Hunan Province
27. ICE of the Second affiliated Hospital of Third Military Medical University
28. ICE of the Affiliated Hospital to Changchun University of Chinese Medicine
29. ICE of the First Hospital of Jilin University
30. Ethics Committee of China-Japan Friendship Hospital Affiliated Jilin University
31. ICE of Jilin Province People’s Hospital
